# A Rare Solitary Fibrous Tumor of the Mesorectum: A Case Report

**DOI:** 10.7759/cureus.58395

**Published:** 2024-04-16

**Authors:** Toshiyuki Moriuchi, Hitoshi Idani, Kohei Taniguchi, Hiroyuki Sawada, Masanori Yoshimitsu

**Affiliations:** 1 Department of Surgery, Hiroshima City Hiroshima Citizens Hospital, Hiroshima, JPN; 2 Department of Pathology, Hiroshima City Hiroshima Citizens Hospital, Hiroshima, JPN

**Keywords:** histopathology, mesenteric tumor, three-dimensional computed tomography, mesorectum, solitary fibrous tumors

## Abstract

Solitary fibrous tumors (SFTs) are rare tumors that predominantly occur in the mesorectum. Few case reports have been published on mesorectal tumors, and this is the seventh case report. A 49-year-old female patient presented with a hypervascularized mesorectal tumor discovered incidentally during a routine medical examination. Using preoperative three-dimensional computed tomography (3D-CT), we identified vessels originating from the superior rectal and lateral sacral arteries, which are important sources of nutrients, and performed the procedure safely and without bleeding. Considering the lack of preoperative diagnosis and rectal blood flow, high anterior resection was performed. The histopathological diagnosis confirmed SFT, and the patient is currently doing well with no recurrence. Although SFT of the mesorectum occurs infrequently, it should be included in the differential diagnosis. In addition, the usefulness of preoperative 3D-CT, including the arterial phase, has been emphasized in such cases. This is the seventh reported case of a rare SFT in the mesorectum. Currently, there is no literature highlighting the usefulness of 3D-CT for SFTs of the mesorectum. However, it is a valuable preparatory tool for preoperative evaluation.

## Introduction

Solitary fibrous tumors (SFTs) were first documented in the pleural region in 1931 [1]. They are uncommon neoplasms with an estimated annual occurrence of 0.35 per 100,000 individuals [2]. This tumor has been observed in various locations, and recently, several cases of abdominopelvic SFTs have been reported [3]. Mesorectal SFTs are rare, and to our knowledge, only six cases have been reported [4-9]. Compared to SFTs in the pleura, SFTs within the pelvic cavity are often larger and are associated with a higher likelihood of malignancy at the time of detection [7,10]. The most efficacious treatment for SFT is surgical excision. A large pelvic SFT generally has large feeding vessels and requires more attention than a small SFT during surgery [11]. In this report, we present a rare case of mesorectal SFT in which preoperative 3D-CT was useful for safe excision.

## Case presentation

**Figure 1:**
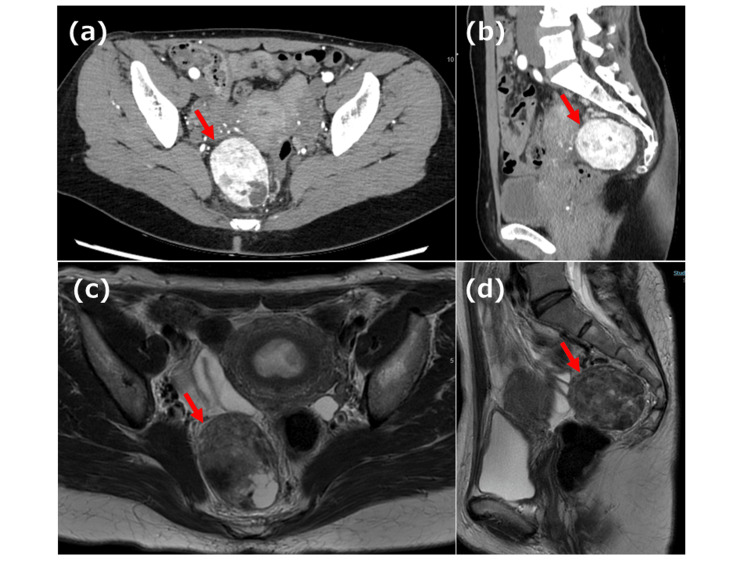
Abdominal computed tomography (CT) and magnetic resonance imaging (MRI) findings a/b: The tumor within the mesorectum is strongly stained on the contrast-enhanced arterial phase CT. The tumor measures 7 × 8 cm. c/d: T2-weighted images showing a heterogeneous lesion with a mixed low-to-high signal.

**Figure 2:**
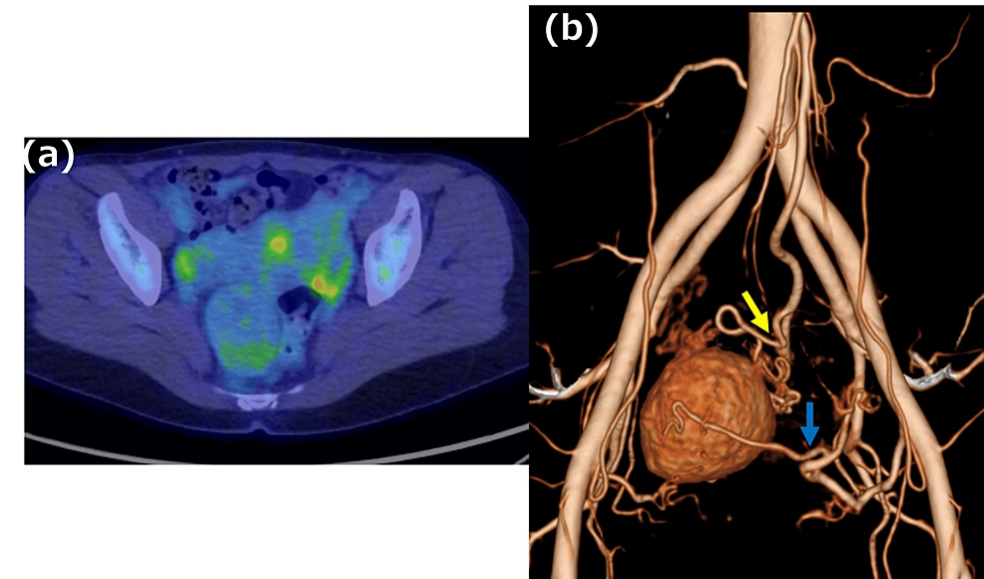
Fluorodeoxyglucose-positron emission tomography/computed tomography and three-dimensional computed tomography findings a: The tumor shows no uptake. b: The tumor has arterial inflow from the superior rectal artery (yellow arrow) and lateral sacral artery (blue arrow).

The patient was a 49-year-old woman with no history of illness, who was referred to our hospital in April 2023 after ultrasonography revealed a tumor in her pelvis. The patient’s serum carcinoembryonic antigen, carbohydrate antigen 19-9, and cancer antigen 125 levels were not elevated. Contrast-enhanced arterial-phase computed tomography (CT) revealed a well-defined, hypervascularized tumor within the mesorectum (Figures 1a, 1b). Pelvic magnetic resonance imaging (MRI) revealed a smooth marginal tumor, and T2-weighted images showed a heterogeneous lesion with a mixed low-to-high signal (Figures 1c, d). Fluorodeoxyglucose positron emission tomography/CT revealed light uptake by the tumor (Figure 2a). Three-dimensional CT (3D-CT) revealed that the tumor's nutrient vessels were the superior rectal and lateral sacral arteries (Figure 2b).

**Figure 3:**
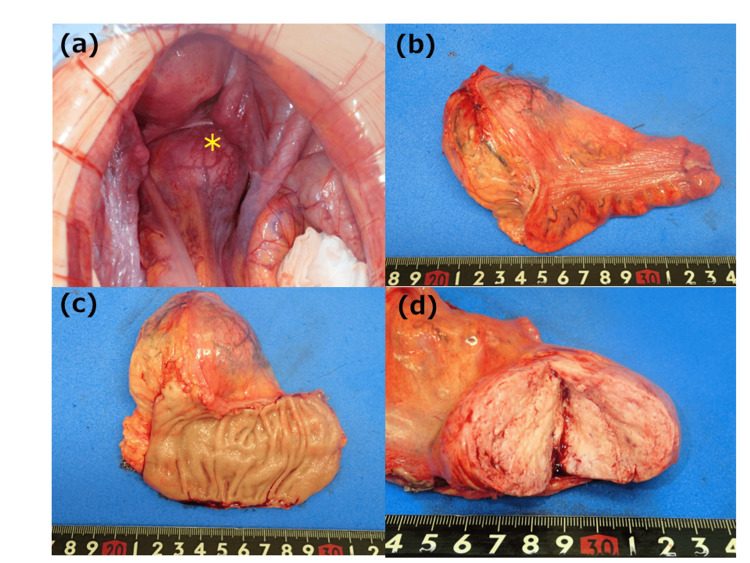
Operative and macroscopic findings a: The tumor has no tendency to invade the surrounding organs. (*tumor) b: The tumor is located in the mesorectum. c: No obvious tendency to invade mucosal surfaces is observed. d: The interior of the tumor is grayish-white and full.

**Figure 4:**
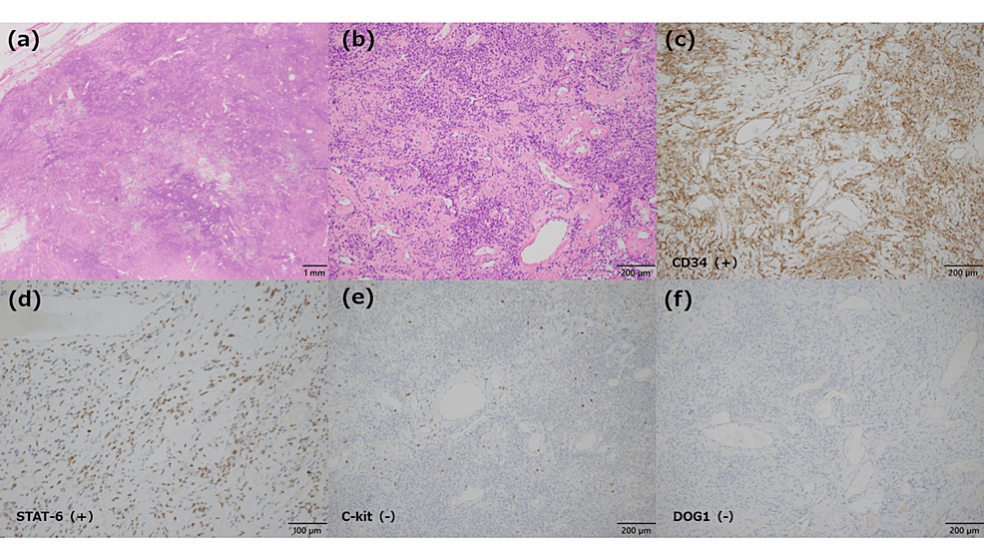
Histopathological findings and presentations of immunohistochemical stains a/b: Hematoxylin and eosin staining reveals spindle tumor cells. Collagen fibrosis and vascular hyperplasia are prominent (original magnification: ×12.5 and ×100). c: CD34 positive (original magnification: ×100). d: STAT6 positive (original magnification: ×200). e: C-kit-negative (original magnification: ×100). f: DOG1 negative (original magnification: ×100).

The tumor was identified as a solid mass in the mesorectum; however, it was judged to be mobile and completely resectable by rectal resection, and a high anterior resection was performed (Figure 3a). There was no invasion of the anterior sacral surface, the total mesorectal excision (TME) layer was maintained and resectable, and the well-developed vein flowing into the tumor was ligated as needed for resection without bleeding. The operative time was 94 min, and minimal blood was lost. The patient had a good postoperative course and was discharged on postoperative day seven. Macroscopic findings showed that the tumor was 80 × 70 × 35 mm in diameter with internal contrast enhancement (Figures 3b-3d). Pathologically, spindle-shaped tumor cells proliferated, and collagen fibers and hypervascularization were prominent (Figures 4a, 4b). The immunohistochemical diagnosis was CD34-positive (Figure 4c), STAT6-positive (Figure 4d), C-kit-negative (Figure 4e), and DOG1-negative (Figure 4f), leading to diagnosis of an SFT. The resection margins were negative, and an R0 resection was completed. The patient had a good postoperative course and remained recurrence-free six months postoperatively.

## Discussion

Mesenchymal tumors were first reported as pleural lesions; however, in recent years, their occurrence at various sites has been increasingly reported. A recent retrospective series showed that 34% of all SFTs occur in the abdominopelvic region, indicating that the abdominopelvic cavity is the primary site of SFT [3]. Among these, SFTs arising from the mesentery are rare, and only six cases to our knowledge have been reported within the mesorectum [4-9]. SFTs are mesenchymal neoplasms. The differential diagnoses included inflammatory pseudotumors, gastrointestinal stromal tumors, leiomyomas, and leiomyosarcomas. As shown in Table 1, preoperative biopsies are rarely available, and preoperative diagnosis is often difficult. In cases where a biopsy is not possible, surgery may be performed for diagnostic or therapeutic purposes.

**Table 1:** Reported cases of mesorectal solitary fibrous tumors

Authors	Year	Sex	Size (cm)	Internal structure	Biopsy	Angiography	Surgical method
Soda et al. [4]	27	F	16×14×9	Heterogeneous enhancement	Yes	Yes	Open rectal resection
Venara et al. [5]	83	M	15	Heterogeneous enhancement	No	No	Open rectal resection
Washiro et al. [6]	65	F	22×15×7	Heterogeneous enhancement	No	Yes	Open low anterior resection
Kawamura et al. [7]	56	F	13×9×7	Heterogeneous enhancement	No	No	Laparoscopic nucleotomy
Ishikawa et al. [8]	35	F	3.7×3.2×3.0	Heterogeneous enhancement	No	No	Laparoscopic nucleotomy
Sekiguchi et al. [9]	64	M	4.0×3.3	Heterogeneous enhancement	No	No	Laparoscopic nucleotomy
Present case	49	F	8×7×5	Heterogeneous enhancement	No	No	Open high anterior resection

Histopathological examination is essential for diagnosis, as it reveals uniform oval spindle-shaped tumors distributed along thin, parallel collagen fibers. Immunohistochemical examination serves as a valuable tool for discerning spindle-shaped tumors that are positive for CD34, STAT6, and Bcl2 [12,13].

Surgical excision with sufficient margins is the optimal approach for managing SFTs. Laparoscopic excision is also undertaken given appropriate margins. Moreover, if blood flow obstruction in the intestinal wall can be avoided, enucleation may be another alternative option. If the tumor displays hypervascularization, intraoperative hemorrhage management becomes imperative. Soda et al. employed aortic balloon catheter insertion for control [4]. Washiro et al. used preoperative angiography to identify blood flow control [6]. Preoperative multi-slice CT or 3D-CT, as illustrated in this instance, may also prove beneficial in identifying the vascular supply. Preoperative dynamic CT may lead to safer surgery, as it allows the surgeon to identify vessels that require intraoperative attention.

Although SFTs are commonly benign, instances of malignancy leading to recurrence have been documented. Elevated recurrence rates correlate with tumor size exceeding 10 cm, a high mitotic rate (≥ 4 mitoses per 10 high-power fields), necrosis, cytological atypia, and positive resection margins [14]. However, clinical behavior cannot always be predicted based on histological features. As recurrence and metastases have been observed even in histologically benign SFTs, strict follow-up is recommended. In this case, no high-risk factors for recurrence were observed; however, a strict follow-up was planned.

## Conclusions

To our knowledge, this is the seventh reported case of a rare SFT in the mesorectum. In cases involving large, hypervascularized tumors, vascular invasion should be anticipated. In this case, the use of preoperative 3D-CT enabled safe surgery. Currently, there is no literature highlighting the usefulness of 3D-CT for SFTs of the mesorectum. However, this is a valuable preparatory tool for preoperative evaluation.
